# Association between the variability of non-high-density lipoprotein cholesterol and the neutrophil-to-lymphocyte ratio in patients with coronary heart disease

**DOI:** 10.3389/fcvm.2023.1254125

**Published:** 2023-11-22

**Authors:** Yifan Chen, Sisi Zhang, Yecheng Tao, Wenyi Hu, Duanbin Li, Xiaohua Shen, Ya Li, Maoning Lin, Wenbin Zhang, Xianglan Liu, DaQi Xie

**Affiliations:** ^1^ Department of Cardiology, Zhongda Hospital, School of Medicine, Southeast University, Nanjing, China; ^2^ Department of Cardiology, Ningbo Ninth Hospital, Ningbo, China; ^3^ Department of Cardiology, Sir Run Run Shaw Hospital, School of Medicine, Zhejiang University, Hangzhou, China; ^4^ Key Laboratory of Cardiovascular Intervention and Regenerative Medicine of Zhejiang Province, Hangzhou, China

**Keywords:** variability, lipid, percutaneous coronary intervention, neutrophil-to-lymphocyte ratio, coronary heart disease

## Abstract

**Background:**

Lowering lipid variability may be a potential strategy for improving the inflammatory state in patients with coronary heart disease (CHD). This study investigated the association between the variability of non-high-density lipoprotein cholesterol (non-HDL-C) and the neutrophil-to-lymphocyte ratio (NLR).

**Methods:**

This study enrolled 2,711 CHD patients subjected to percutaneous coronary intervention (PCI). During the 1-year follow-up period after PCI, the variability of non-HDL-C was assessed using standard deviation (SD), coefficient of variation (CV), and variability independent of mean (VIM). NLR was calculated as the ratio of absolute neutrophil count to absolute lymphocyte count. The relationship between the non-HDL-C variability and the average NLR level during follow-ups was examined using a linear regression analysis.

**Results:**

The mean age of the patients was 64.4 ± 10.8 years, with 72.4% being male. The average NLR level was 2.98 (2.26–4.14) during the follow-up (1 year after PCI). The variability of non-HDL-C was 0.42 (0.26–0.67) for SD, 0.17 (0.11–0.25) for CV, and 0.02 (0.01–0.03) for VIM. A locally weighted scatterplot smoothing curve indicates that the average levels of NLR increased with increasing variability of non-HDL-C. Regardless of the variability assessment method used, non-HDL-C variability was significantly positively associated with the average NLR level during follow-ups: SD [*β* (95% CI) = 0.681 (0.366–0.996)], CV [*β* (95% CI) = 2.328 (1.458–3.197)], and VIM [*β* (95% CI) = 17.124 (10.532–23.715)]. This association remained consistent across subgroups stratified by age, gender, diabetes, and hypertension.

**Conclusion:**

The variability of non-HDL-C was positively associated with NLR in patients with CHD, suggesting that reducing non-HDL-C variability may improve the low-grade inflammatory state in CHD patients.

## Background

Coronary heart disease (CHD) is a major health concern that significantly impacts the quality of life of the patients (1). The pathogenesis of atherosclerosis, the primary underlying cause of CHD, is driven by the fundamental theories of lipid deposition and inflammation (1).

Numerous clinical trials have provided substantial evidence supporting the role of lowering low-density lipoprotein cholesterol (LDL-C) in reducing the development of atherosclerotic plaques and thereby mitigating the incidence of cardiovascular diseases (2–4). Despite achieving the target LDL-C level following the lipid-lowering therapy, CHD patients may still be exposed to a substantial risk of cardiovascular diseases (5). This suggests that LDL-C alone is not the sole determinant of atherosclerotic cardiovascular disease (ASCVD) risk. In recent years, non-high-density lipoprotein cholesterol (non-HDL-C), which encompasses all cholesterol except HDL-C, has garnered increasing attention in research studies (6). A meta-analysis of statin-treated patients has demonstrated that non-HDL-C levels exhibit a superior predictive ability for future major cardiovascular events compared with LDL-C levels (7). Several guidelines also suggest targeting non-HDL-C as a secondary approach in preventing and treating ASCVD (7).

The inflammatory response plays a persistent role in the pathogenesis of CHD and atherosclerosis. While C-reactive protein (CRP) serves as the established marker of inflammation, there is an increasing recognition of the neutrophil-to-lymphocyte ratio (NLR). The NLR, derived from peripheral blood, is a significant indicator of inflammation, reflecting the balance between neutrophils and lymphocytes. Neutrophils are associated with non-specific inflammation processes, whereas lymphocytes indicate immune regulation. A decrease in lymphocyte levels has been linked to the progression of atherosclerosis (8). Moreover, elevated levels of NLR have been identified as prognostic markers for cardiovascular disease (9).

Numerous studies have demonstrated a strong link between lipid metabolism abnormalities and inflammatory conditions, but they primarily focus on the absolute levels of blood lipids (10, 11). The variability of lipids could provide an alternative characteristic of lipids in patients with CHD. Our previous research revealed that variability in the serum levels of HDL-C and LDL-C is predictive of NLR, an inflammatory indicator (12). However, limited attention has been given to the variability of non-HDL-C.

This study aimed to investigate the association between the variability of non-HDL-C and the average level of NLR during the 1-year follow-up period in patients with CHD after undergoing percutaneous coronary intervention (PCI).

## Methods

### Study population

This retrospective, multicenter observational study was conducted at Sir Run Run Shaw Hospital and its medical consortium hospitals. Eligible CHD patients who received PCI between 2010 and 2019 were systematically enrolled. The inclusion criteria required a diagnosis of CHD with elective PCI, a minimum of three visits during the first-year follow-ups, and comprehensive information about baseline and follow-ups. The exclusion criteria included severe valvular heart disease, peripheral artery disease, congenital heart disease, heart failure with New York Heart Association (NYHA) class IV, hematological disorders, malignant tumors, severe liver and kidney dysfunction, immunological disorder, and severe acute/chronic infection. Skilled interventional cardiologists performed all PCI procedures in accordance with the current guidelines (13) using either the femoral or radial artery approach. All patients underwent their initial PCI and commenced lipid-lowering therapy in the perioperative period to maintain a consistent lipid-lowering regimen throughout the 1-year follow-up period. The Ethics Committee of Sir Run Run Shaw Hospital of Zhejiang University approved the current study (No. 20201217-36).

### Neutrophil-to-lymphocyte ratio

Baseline information was obtained by collecting blood samples from the patients 24 h before undergoing PCI. Follow-ups were scheduled in the first year after PCI, and at least three follow-up visits were conducted. Blood samples were taken from the anterior cubital vein after an overnight fast to perform routine laboratory assessments. The counts of neutrophils and lymphocytes in blood were analyzed using an automated blood cell counter. The NLR was calculated as the ratio of the absolute neutrophil count to the absolute lymphocyte count.

### Variability of non-HDL-C

Lipid measurements, including total cholesterol (TC), triglycerides (TGs), LDL-C, HDL-C, and very-low-density lipoprotein (VLDL) cholesterol, were performed using a blood chemistry analyzer (Hitachi 747, Tokyo, Japan).

The variability of non-HDL-C was evaluated using three methods: (1) standard deviation (SD) method, the standard deviation of multiple measurements of non-HDL-C during the follow-up; (2) coefficient of variation (CV) method, CV = (SD/mean) × 100(%); and (3) variability independent of mean (VIM) method, VIM = (SD/mean*^β^*) × 100(%), where *β* is derived from curve fitting based on coefficients of the natural logarithm of SD (14). Based on the variability of non-HDL-C, the participants were categorized into high-, medium-, and low-variability groups.

### Definition of covariates

The Health Information System (HIS) provides data on patient demographics and blood biochemistry tests. Patients who were smokers or had quit smoking within 3 months were classified as having a smoking history. Hypertension was defined as three separate instances of diastolic blood pressure at ≥90 mmHg and/or systolic blood pressure at ≥140 mmHg in the absence of antihypertensive medication. Diabetes was diagnosed based on typical symptoms of diabetes (polydipsia, polyuria, unexplained weight loss) with a fasting blood glucose of ≥7.0 mmol/L or a random blood glucose level of ≥11.1 mmol/L.

### Statistical analysis

Normally distributed continuous variables were expressed as mean ± standard deviation and compared using the *t*-test. Non-normally distributed continuous variables were expressed as median (interquartile range) and compared using the Mann–Whitney *U* test. Categorical variables were expressed as counts (percentages) and compared using the chi-squared test.

A locally weighted scatterplot smoothing (LOESS) curve (span = 1) was employed to depict the relationship between the variability of non-HDL-C and the average level of NLR. The Spearman correlation test was used to assess the correlation among the non-HDL-C variability and the NLR level, with the correlation coefficient *ρ* being shown.

A linear regression model was used to assess the association between the variability of non-HDL-C and the average level of NLR during follow-ups. The covariates with a univariable analysis (*P*-value < 0.1) were further adjusted in multivariable regression analysis. Restricted cubic spline analysis with four knots was used to assess the association between the variability of non-HDL-C and the high level of NLR (average NLR of >3), with variability distributions outside the range of 5%–95% being excluded. The covariates with a univariable analysis (*P*-value < 0.1) were also adjusted in the restricted cubic spline analysis. Subgroup analyses were conducted by using the multivariable linear regression model in patients stratified by age (≥65 or <65 years old), gender (male or female), diabetes (presence or absence), and hypertension (presence or absence).

All statistical analyses were conducted using R software. A significance level of *P* < 0.05 was considered statistically significant.

## Results

### Baseline characteristic

This study included a total of 2,711 CHD participants who received elective PCI. The mean age of the participants was 64 years, with 72% being male. Among the patients, 27% reported smoking, 65% had hypertension, and 25% had diabetes. At baseline, the mean levels of NLR and non-HDL-C were 2.63 (1.94–3.96) and 3.32 ± 1.20 mmol/L, respectively. During follow-ups, the mean levels of NLR and non-HDL-C were 2.98 (2.26–4.14) and 2.59 ± 0.74 mmol/L, respectively. Detailed population characteristics, such as demographic information, laboratory test results, and medication details, are presented in Table 1.

**Table 1:** Population characteristics at baseline and follow-ups.

	Overall (*N* = 2,711)	Variability VIM		*P-*value
		Low group (<0.0166, *N* = 1,356)	High group (≥0.0166, *N* = 1,355)	
Demographic				
Age (years)	64.43 (10.83)	65.01 (10.52)	63.85 (11.10)	0.005
Male (%)	1,964 (72.4)	995 (73.4)	969 (71.5)	0.297
BMI (kg/m^2^)	24.52 (3.00)	24.53 (2.97)	24.51 (3.03)	0.920
Currently smoking (%)	741 (27.3)	366 (27.0)	375 (27.7)	0.721
Diabetes (%)	688 (25.4)	328 (24.2)	360 (26.6)	0.168
Hypertension (%)	1,758 (64.8)	921 (67.9)	837 (61.8)	0.001
Prior PCI (%)	184 (6.8)	103 (7.6)	81 (6.0)	0.110
Baseline data				
Non-HDL-C (mmol/L)	3.32 (1.20)	3.05 (1.06)	3.58 (1.27)	<0.001
NLR	2.63 (1.94–3.96)	2.61 (1.90–3.81)	2.67 (1.98–4.13)	0.026
Hemoglobin (g/dl)	13.24 (1.73)	13.08 (1.66)	13.39 (1.78)	<0.001
eGFR (ml/min/1.73 m^2^)	98.09 (34.10)	96.76 (31.28)	99.43 (36.67)	0.041
NT-proBNP (ng/ml)	0.17 (0.05–0.74)	0.17 (0.05–0.73)	0.17 (0.06–0.77)	0.195
UA (μmol/dl)	36.56 (9.67)	36.53 (9.58)	36.58 (9.75)	0.902
FBG (mmol/L)	6.72 (2.69)	6.50 (2.36)	6.94 (2.98)	<0.001
Follow-up data				
Average Non-HDL-C (mmol/L)	2.59 (0.74)	2.51 (0.71)	2.67 (0.76)	<0.001
Average NLR	2.98 (2.26–4.14)	2.92 (2.19–3.99)	3.07 (2.29–4.39)	0.002
Non-HDL-C SD	0.42 (0.26–0.67)	0.26 (0.18–0.35)	0.66 (0.51–0.89)	<0.001
Non-HDL-C CV	0.17 (0.11–0.25)	0.11 (0.08–0.14)	0.25 (0.21–0.32)	<0.001
Non-HDL-C VIM	0.02 (0.01–0.03)	0.01 (0.01–0.01)	0.03 (0.02–0.04)	<0.001
Average HDL-C (mmol/L)	1.03 (0.25)	1.04 (0.25)	1.03 (0.25)	0.436
Average LDL-C (mmol/L)	1.88 (0.62)	1.83 (0.60)	1.94 (0.63)	<0.001
Average WBC (×10^3^/L)	6.41 (1.28)	6.33 (1.27)	6.48 (1.29)	0.002
Average CRP (mg/L)	1.54 (0.81–2.850	1.50 (0.78–2.72)	1.63 (0.84–2.90)	0.050
Medication (%)				
ACEI or ARB	1,651 (60.9)	838 (61.8)	813 (60.0)	0.357
Beta-blocker	1,628 (60.1)	837 (61.7)	791 (58.4)	0.082
CCB	799 (29.5)	428 (31.6)	371 (27.4)	0.019
Statin	2,663 (98.2)	1,341 (98.9)	1,322 (97.6)	0.013
Intensive statin	469 (17.3)	215 (15.9)	254 (18.7)	0.053
Ezetimibe	427 (15.8)	143 (10.5)	284 (21.0)	<0.001

BMI, body mass index; PCI, percutaneous coronary intervention; non-HDL-C, non-high-density lipoprotein cholesterol; NLR, neutrophil-to-lymphocyte ratio; SD, standard deviation; CV, coefficient of variation; VIM, variability independent of mean; eGFR, estimated glomerular filtration rate; NT-proBNP, N-terminal pro-B-type natriuretic peptide; UA, uric acid; FBG, fasting blood glucose; WBC, white blood cell count; CRP, C-reactive protein; ACEI, angiotensin-converting enzyme inhibitors; ARB, angiotensin II receptor blocker; CCB, calcium channel blocker.

Continuous variables are expressed as mean ± SD or median (IQR), whereas categorical variables are presented as counts (percentages). Patients were equally divided into low- and high-variability groups based on VIM (cutoff value = 0.0166) during the follow-up period.

### Trend in average NLR according to non-HDL-C variability

**Figure 1:**
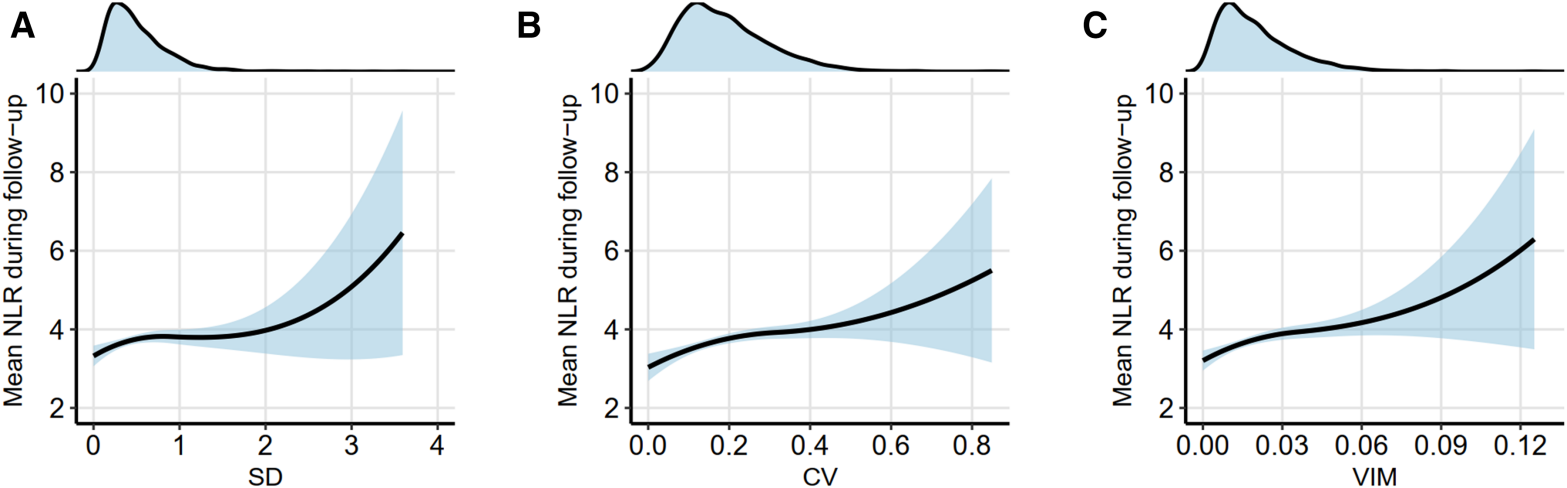
Trend in the average level of NLR according to non-HDL-C variability. A locally weighted scatterplot smoothing (LOESS) curve (span = 1) was employed to depict the trend in the average level of NLR according to non-HDL-C variability. Non-HDL-C variability was assessed using three measures: standard deviation (**A**), coefficient of variation (**B**), and variability independent of the mean (**C**). The upper density plot illustrates the distribution of lipid variability among patients. Refer to Table 1 for abbreviations.

The LOESS curve (span = 1) depicts the trend in the average level of NLR according to non-HDL-C variability. In Figure 1, the average levels of NLR increased with increasing non-HDL-C variability, regardless of the method used for variability assessment. Density plots indicate a right-skewed distribution of non-HDL-C variability.

### Correlation matrix among non-HDL-C variability and levels

**Figure 2:**
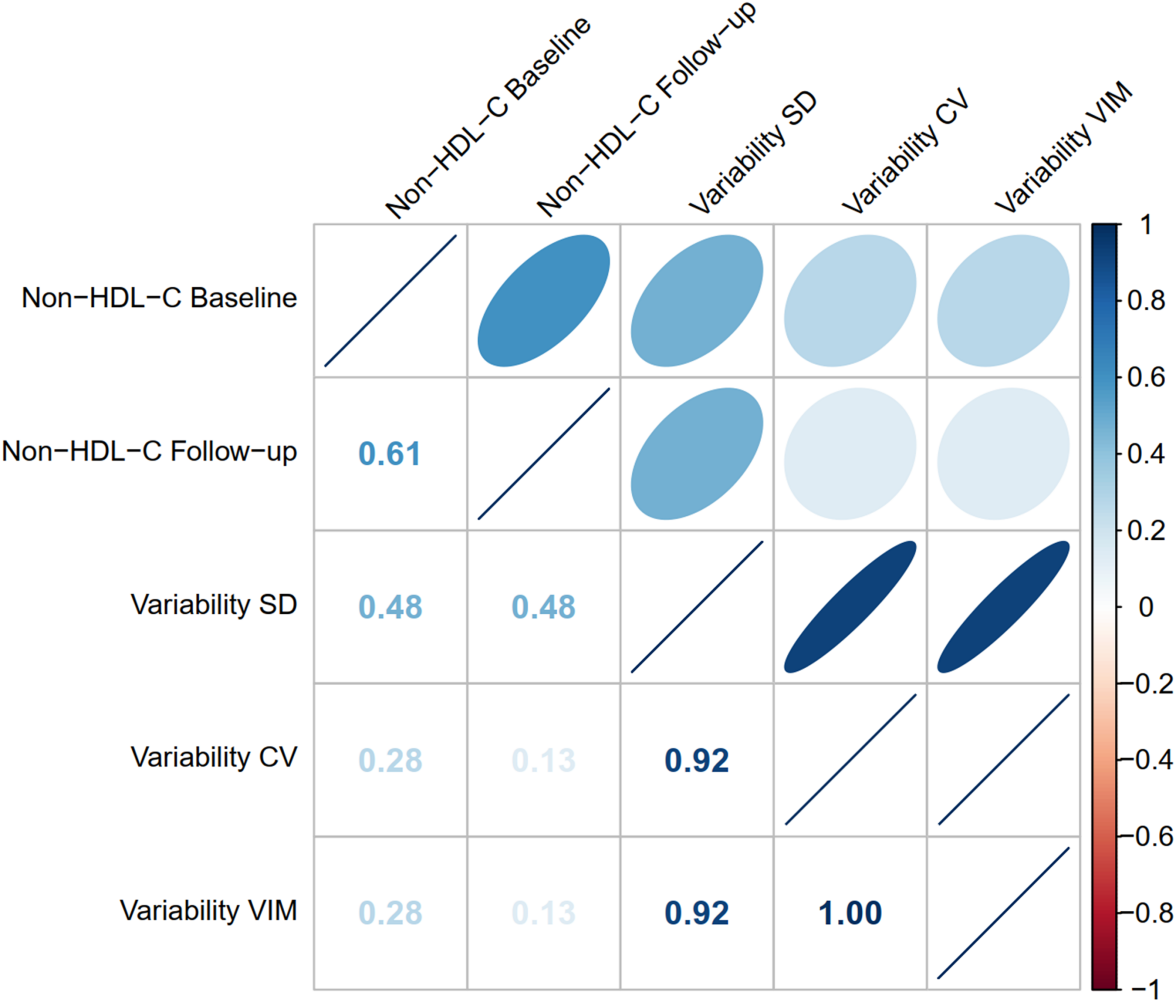
Correlation matrix among non-HDL-C variability and levels. The Spearman method was used to assess the correlation among non-HDL-C variability and levels. The P-values for all Spearman correlation tests were less than 0.05. The correlation coefficient ρ is shown on the lower left. When ρ is +1, it indicates a perfectly positive correlation; when ρ is −1, it indicates a perfectly negative correlation. Refer to Table 1 for abbreviations

In the correlation matrix (Figure 2), all *P*-values for the Spearman tests were less than 0.05. During follow-ups, the average level of non-HDL-C was positively associated with non-HDL-C variability for SD (*ρ* = 0.48), CV (*ρ* = 0.13), and VIM (*ρ* = 0.13), whereas the three variability indicators were highly correlated with each other (SD and CV: *ρ* = 0.92; SD and VIM: *ρ* = 0.92; CV and VIM: *ρ* = 1.00).

### Association between bon-HDL-C variability and average NLR levels

Univariable linear regression analysis revealed that several patient characteristics (confounders), such as age, gender, BMI, diabetes, hypertension, history of PCI, follow-up levels of non-HDL-C and HDL-C, hemoglobin, estimated glomerular filtration rate (eGFR), NT-proBNP, uric acid, fasting blood glucose, beta-blocker, statin, ezetimibe, and intensive statin, were associated with the average levels of NLR (*P*-value < 0.1). These confounders were thus adjusted in the multivariable regression model. In Table 2, the results suggested that increased variability of non-HDL-C was associated with a higher average level of NLR, regardless of the non-HDL-C variability assessed using SD [*β* (95% CI) = 0.681 (0.366–0.996)], CV [*β* (95% CI) = 2.328 (1.458–3.197)], or VIM [*β* (95% CI) = 17.124 (10.532–23.715)].

**Table 2:** Linear regression analyses between the variability of non-HDL-C and the average level of NLR during follow-ups.

Variables	Univariable			Multivariable (SD)			Multivariable (CV)			Multivariable (VIM)		
	Beta	95% CI	*P*-value	Beta	95% CI	*P*-value	Beta	95% CI	*P*-value	Beta	95% CI	*P*-value
Non-HDL-C SD	0.173	−0.114 to 0.46	0.237	0.681	0.366 to 0.996	<0.001						
Non-HDL-C CV	1.788	0.874 to 2.702	<0.001				2.328	1.458 to 3.197	<0.001			
Non-HDL-C VIM	13.045	6.108 to 19.982	<0.001							17.124	10.532 to 23.715	<0.001
Age (years)	0.045	0.036 to 0.054	<0.001	0.033	0.023 to 0.042	<0.001	0.033	0.023 to 0.042	<0.001	0.033	0.023 to 0.042	<0.001
Male	0.387	0.164 to 0.61	0.001	0.503	0.269 to 0.738	<0.001	0.518	0.284 to 0.752	<0.001	0.518	0.284 to 0.753	<0.001
BMI (kg/m^2^)	−0.091	−0.124 to −0.058	<0.001	−0.091	−0.124 to −0.058	<0.001	−0.091	−0.123 to −0.058	<0.001	−0.091	−0.124 to −0.058	<0.001
Smoking	−0.139	−0.363 to 0.085	0.223									
Diabetes	0.227	−0.002 to 0.456	0.053	−0.196	−0.432 to 0.04	0.103	−0.199	−0.435 to 0.037	0.098	−0.199	−0.434 to 0.037	0.099
Hypertension	0.238	0.029 to 0.447	0.026	0.146	−0.057 to 0.348	0.159	0.151	−0.05 to 0.353	0.142	0.15	−0.052 to 0.352	0.145
Prior PCI	0.371	−0.026 to 0.768	0.067	0.302	−0.062 to 0.667	0.104	0.313	−0.05 to 0.677	0.091	0.311	−0.053 to 0.675	0.094
Average non-HDL-C	−0.202	−0.337 to −0.067	0.003	−0.263	−0.409 to −0.117	<0.001	−0.13	−0.257 to −0.002	0.046	−0.128	−0.255 to 0	0.05
Average HDL-C	−1.995	−2.384 to −1.606	<0.001	−1.99	−2.379 to −1.6	<0.001	−1.985	−2.374 to −1.596	<0.001	−1.983	−2.372 to −1.594	<0.001
Hemoglobin (g/dl)	−0.217	−0.274 to −0.16	<0.001	−0.096	−0.16 to −0.032	0.003	−0.102	−0.166 to −0.038	0.002	−0.101	−0.165 to −0.037	0.002
eGFR (ml/min/1.73 m^2^)	−0.011	−0.014 to −0.008	<0.001	−0.004	−0.007 to 0	0.023	−0.004	−0.007 to 0	0.024	−0.004	−0.007 to 0	0.024
NT-proBNP (ng/ml)	0.26	0.222 to 0.298	<0.001	0.161	0.122 to 0.201	<0.001	0.16	0.121 to 0.2	<0.001	0.161	0.121 to 0.2	<0.001
UA (μmol/dl)	0.019	0.009 to 0.029	<0.001	0.001	−0.01 to 0.011	0.913	0	−0.011 to 0.011	0.972	0	−0.011 to 0.011	0.97
FBG (mmol/L)	0.096	0.059 to 0.133	<0.001	0.076	0.037 to 0.114	<0.001	0.074	0.036 to 0.112	<0.001	0.074	0.036 to 0.112	<0.001
ACEI or ARB	0.038	−0.167 to 0.243	0.716									
Beta-blocker	−0.248	−0.452 to −0.044	0.017	−0.095	−0.286 to 0.096	0.33	−0.093	−0.284 to 0.098	0.339	−0.094	−0.285 to 0.096	0.333
CCB	−0.128	−0.347 to 0.091	0.252									
Statin	−3.563	−4.309 to −2.817	<0.001	−3.082	−3.783 to −2.381	<0.001	−3.049	−3.749 to −2.349	<0.001	−3.058	−3.758 to −2.358	<0.001
Ezetimibe	−0.274	−0.548 to 0	0.05	−0.078	−0.337 to 0.18	0.552	−0.108	−0.366 to 0.151	0.414	−0.105	−0.363 to 0.154	0.428
Intensive statin	−0.229	−0.493 to 0.035	0.089	−0.156	−0.402 to 0.09	0.215	−0.165	−0.41 to 0.081	0.19	−0.162	−0.408 to 0.084	0.198

In multivariable regression analysis, covariates with a univariable analysis (*P*-value < 0.1) were further adjusted. Refer to Table 1 for other abbreviations.

### Association between non-HDL-C variability and high inflammatory state

**Figure 3:**
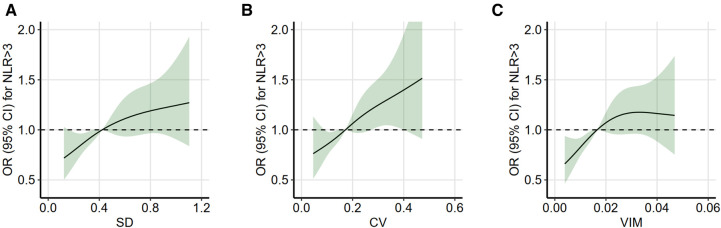
Restricted cubic spline analysis between non-HDL-C variability and high inflammatory state. Restricted cubic spline analysis with four knots was used to assess the association between non-HDL-C variability and high inflammatory state (average NLR of >3), which was based on the logistic regression model. Non-HDL-C variability was assessed using three measures: standard deviation (**A**), coefficient of variation (**B**), and variability independent of the mean (**C**). Variability distributions outside the range of 5%–95% were considered as potential outliers and excluded. The model was adjusted for the significant confounders (*P-*value of <0.1) identified in the univariable analysis (in Table 2). OR indicates odds ratio; refer to Table 1 for other abbreviations.

A high inflammatory state is defined as an average NLR level of >3. Based on the adjusted logistic regression model, the restricted cubic spline plot (four knots) reveals that the risk of a high inflammatory state increases with non-HDL-C variability (Figure 3). This association is consistent across the three variability assessment methods. These models were adjusted for potential confounders identified in Table 2 and excluded potential outliers with variability distributed outside the 5%–95% range.

### Subgroup analysis

**Figure 4:**
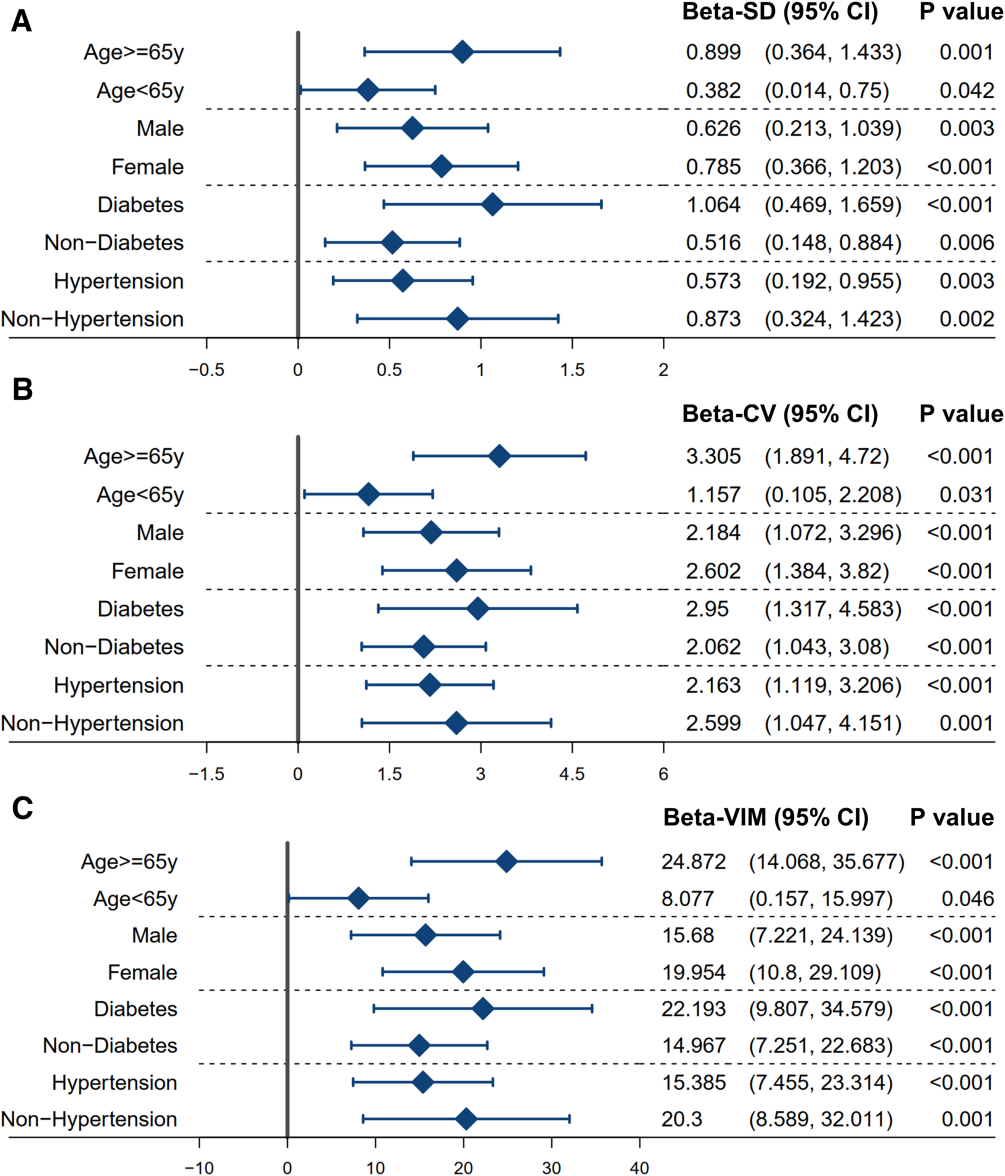
Subgroup analysis between the variability of non-HDL-C and the average level of NLR. The linear regression model was used to assess the association between the variability of non-HDL-C and the average level of NLR in a stratified population. The model was adjusted for the significant confounders (*P*-value of <0.1) identified in the univariable analysis (in Table 2). The variability of non-HDL-C is measured using the standard deviation (**A**), coefficient of variation (**B**), and variability independent of the mean (**C**). Refer to Table 1 for abbreviations.

Based on the linear regression model, subgroup analysis confirmed a consistent result with the main findings in patients stratified by age (<65 or ≥65 years), gender (male or female), diabetes mellitus (no or yes), and hypertension (no or yes) (Figure 4). The *P*-values of the regression analysis for all subgroups were less than 0.05. The robustness of this result was also assessed using different variability assessment methods, such as SD, CV, and VIM.

## Discussion

This study identified a significant association between the variability of non-HDL-C and the average level of NLR during the 1-year follow-up of elective PCI patients, irrespective of age, gender, hypertension, or diabetes.

Atherosclerosis is fundamentally influenced by lipid levels (15). The non-HDL-C and LDL-C parameters have been implicated in the pathogenesis and progression of cardiovascular complications, atherosclerosis, and chronic inflammation (16, 17). Non-HDL-C is a more direct and accurate marker of all atherogenic lipoprotein particles compared with LDL-C. The Bypass Angioplasty Revascularization Investigation (BARI) study found non-HDL-C to be the most traditional lipid parameter with the most prognostic predictive value for CHD patients during 5-year follow-ups (18). In patients with hypertriglyceridemia, the level of LDL-C could be underestimated as a result of enhanced exchange, whereas the levels of non-HDL-C remain unaffected, providing a continuous risk estimate (19). Hence, non-HDL-C is a superior metric for tracking atherosclerotic lipid indicators and overall lipid status during follow-up.

Most research primarily considers the absolute value of the lipid metabolism index, neglecting its variability. However, recent evidence indicates that lipid metabolism variability is equally important to its average level. Previous research had linked LDL-C variability to inflammation (12), whereas another study identified LDL-C variability as a key factor in coronary atherosclerosis progression (20). The variability in the remaining lipids, for instance, Lp(a), TG, and VLDL, has a significant impact on post-PCI CHD patients. This investigation emphasizes non-HDL-C as a comprehensive measure of lipid metabolism characteristics, revealing the correlation between atherogenic cholesterol and inflammatory status. We discovered a significant correlation between the variability of non-HDL-C and the level of NLR, suggesting non-HDL-C variability as an independent predictor of chronic inflammatory status in post-PCI CHD individuals (21).

The specific mechanism by which increased non-HDL-C variability promotes inflammation remains unclear. However, lowering the lipid variability could potentially affect the level of NLR and improve post-PCI patient prognosis. LDL-C, Lp(a), and VLDL in blood might infiltrate and oxidize the arterial wall endothelium, inducing inflammation and endothelial damage. Increased variability of non-HDL-C possibly destabilizes plaque stability mechanisms, resulting in pro-inflammatory factor release and plaque vulnerability. In addition, high variability could indicate a longer duration in which lipids are outside the targeted range, leading to worsened prognosis. Other metabolic and genetic mechanisms may also be involved, such as polymorphism in 3-hydroxy-3-methylglutaryl coenzyme A (HMG-CoA) reductase, VLDL receptor, and LDL-C receptor (22–25). This study observed an underlying influence of non-HDL-C variability on NLR, an inflammatory indicator, in post-PCI CHD patients, irrespective of age, sex, hypertension, and diabetes. The three variability analysis methods employed in this study revealed a robust link, emphasizing the significant, stable inflammatory feedback effect of non-HDL-C unaffected by other factors.

The well-established association between NLR and adverse outcomes in patients undergoing PCI is noteworthy. For instance, Hong et al. (26) demonstrated that among individuals with acute myocardial infarction undergoing PCI, a high level of NLR post-PCI was linked to an elevated risk of large-sized infarctions and unfavorable clinical outcomes. Furthermore, a meta-analysis (27) has corroborated NLR as a predictive factor for both hospitalization rates and long-term prognosis in patients experiencing acute ST-segment elevation myocardial infarction post-PCI. In addition, Kim et al. (28) provided compelling evidence that a high level of NLR reliably predicts cardiac mortality following PCI, particularly in patients with pre-existing heart failure or myocardial injury. These collective findings underscore the significant prognostic value of NLR in evaluating the outcomes of coronary artery disease (CAD) patients undergoing PCI.

This study contributes several notable strengths to the field of cardiovascular research. First, it sheds light on an understudied dimension of non-HDL-C variability and its relationship with inflammation levels, as measured by NLR, in patients undergoing elective PCI. By focusing on non-HDL-C as a comprehensive lipid measure, the study expands our understanding beyond traditional markers like LDL-C, providing a more comprehensive assessment of atherogenic lipoprotein particles. Moreover, the analysis considers the variability and stability of lipid levels, moving beyond average values and deepening our knowledge of lipid metabolism in cardiovascular health. In addition, the study encompasses diverse patient subgroups, such as age, gender, and individuals with hypertension and diabetes, thus enhancing the generalizability of the findings. Finally, the inclusion of three different methods for variability analysis strengthens the robustness of the results. This innovative approach may pave the way for future research, encouraging exploration beyond conventional lipid measures and emphasizing the significance of lipid variability in studying cardiovascular diseases.

Among patients undergoing PCI, Lee et al. conducted a study investigating the relationship between non-HDL-C variability and cardiovascular outcomes (29). Notably, the patient characteristics in their study closely resemble those in our cohort [mean follow-up non-HDL-C: 2.72 ± 0.69 vs. 2.59 ± 0.74 mmol/L; visit-to-visit non-HDL-C variability (standard deviation): 0.44 vs. 0.42 mmol/L]. The Kaplan–Meier analysis revealed a significant increase in the risk of major adverse cardiovascular events in the highest quartile (Q4) compared with the lower three quartiles (Q1–Q3). Based on these findings, we posit that non-HDL-C variability should be restricted to levels below the 75th percentile within the PCI population to mitigate the risk of cardiovascular events.

Despite its contributions, this study has several limitations. First, its retrospective design may have introduced a selection bias. Second, the focus on post-PCI inflammation levels without follow-up on endpoint event outcomes limits the comprehensive assessment of the study. Third, using NLR as an inflammatory indicator may not capture the full complexity of the inflammatory response. Future studies incorporating molecular biology techniques to evaluate additional inflammatory markers such as tumor necrosis factors and interleukins would provide a more comprehensive understanding. Fourth, some characteristics of lipid-lowering therapy that are difficult to assess may still influence the variability of lipids. Finally, the relatively short follow-up period may not capture long-term interactions between lipid metabolism and inflammation. To address these limitations, future prospective randomized controlled trials with larger sample sizes are recommended.

## Conclusion

The variability of non-HDL-C is positively associated with NLR in patients with CHD, suggesting that reducing non-HDL-C variability may improve the low-grade inflammatory state in patients with CHD.

## Data Availability

The raw data supporting the conclusions of this article will be made available by the authors, without undue reservation.
